# Commentary on “Imaging-based surrogate classification for risk stratification of hepatocellular carcinoma with microvascular invasion to adjuvant hepatic arterial infusion chemotherapy: a multicenter retrospective study”

**DOI:** 10.1097/JS9.0000000000003350

**Published:** 2025-09-08

**Authors:** Weiyu Zhang, Yutong Chen

**Affiliations:** a Center of Vascular Surgery & Interventional Radiology, Shengli Clinical Medical College of Fujian Medical University, Fuzhou University Affiliated Provincial Hospital, School of Medinine, Fuzhou University, Fuzhou City, Fujian Province, China; b Department of Oncology, Shengli Clinical Medical College of Fujian Medical University, Fuzhou University Affiliated Provincial Hospital, School of Medinine, Fuzhou University, Fuzhou City, Fujian Province, China

*Dear Editor,*

We read with great interest the recent study by Ma *et al*, which proposed an imaging-based prognostic model aimed at stratifying patients with microvascular invasion (MVI)-positive hepatocellular carcinoma (HCC) and guiding the use of adjuvant hepatic arterial infusion chemotherapy (HAIC)^[1]^. While the study addresses a clinically relevant and timely topic, we would like to raise several concerns regarding the study design, statistical interpretation, and the practical applicability of the findings.

First, the four final imaging features included in the nomogram arterial peritumoral enhancement, tumor enhancement margin, layered necrosis, and necrotic area boundary were selected from a set of potentially collinear variables through conventional regression modeling. Such manual feature engineering may be subject to observer bias and inter-reader variability, despite the reported agreement statistics being statistically significant. Integrating reproducible, automated radiomics pipelines that include feature extraction and repeatability filtering could improve objectivity and standardization across institutions.

Second, alternative modeling approaches such as penalized regression (LASSO-Cox), survival random forests, or deep learning-based survival models (DeepSurv) are capable of capturing nonlinear interactions and handling high-dimensional data inputs^[2]^. These methods may yield better calibration and discrimination, particularly in external validation cohorts^[3]^. Given that the study involved CT and MRI data from multiple centers, deep learning models pre-trained on large datasets and fine-tuned on institution-specific data may be particularly well-suited to handling such heterogeneous imaging inputs.

Although the proposed model demonstrated good discrimination (AUC 0.73–0.84), its incremental value over established clinical-pathological predictors remains to be fully elucidated. Notably, several conventional prognostic factors for HCC recurrence such as tumor size, presence of satellite nodules, and AFP levels^[4]^ were not included in the final model. A rationale for focusing solely on imaging features is needed, especially when combined models may offer superior predictive accuracy.

It is also worth highlighting that the current model did not incorporate multimodal or quantitative imaging data, despite growing interest in integrating radiological features with genomic information to enhance biological interpretability. As the authors rightly point out, RNA sequencing analysis in their study identified immune-regulatory pathways associated with imaging features. This underscores the value of building integrated pipelines that combine radiomics with transcriptomic or immunologic data to better capture tumor microenvironment heterogeneity and treatment response. However, these associations remain descriptive and require further functional validation to substantiate their clinical relevance.

In summary, we commend the authors for their innovative efforts to improve risk stratification in MVI-positive HCC. We believe that future prospective validations, assessments of the model’s utility in predicting HAIC benefit, and integration of multimodal clinical, imaging, and molecular data will be critical steps toward facilitating the model’s clinical translation. The above content complies with the TITAN guidelines^[5]^.

## Data Availability

Not applicable.
